# Supplementary material for: Nonlocal meta-lens with Huygens’ bound states in the continuum
Source: Nat Commun. 2024 Aug 2;15:6543. doi: 10.1038/s41467-024-50965-y (PMC11297327; doi:10.1038/s41467-024-50965-y)
Supplement: Supplementary file 1 — Supplementary Information [file 41467_2024_50965_MOESM1_ESM.pdf]

Supplementary Information

Nonlocal meta-lens with Huygens' bound states in the continuum

Jin Yao,[1,†] Fangxing Lai,[2,†] Yubin Fan,[1,†] Yuhan Wang,[2] Shih-Hsiu Huang,[3] Borui Leng,[1] Yao Liang,[1] Rong Lin,[1] Shufan Chen,[1] Mu Ku Chen,[1,4,5,*] Pin Chieh Wu,[3,6,7,*] Shumin Xiao,[2,*] and Din Ping Tsai[1,4,5,*]

[1] *Department of Electrical Engineering, City University of Hong Kong, Kowloon, Hong Kong SAR, China*

[2] *State Key Laboratory on Tunable Laser Technology, Ministry of Industry and Information Technology Key Lab of Micro-Nano Optoelectronic Information System, Shenzhen Graduate School, Harbin Institute of Technology, Shenzhen 518055, China*

[3] *Department of Photonics, National Cheng Kung University, Tainan 70101, Taiwan*

[4] *Centre for Biosystems, Neuroscience, and Nanotechnology, City University of Hong Kong, Kowloon, Hong Kong SAR, China*

[5] *State Key Laboratory of Terahertz and Millimeter Waves, City University of Hong Kong, Kowloon, Hong Kong SAR, China*

[6] *Center for Quantum Frontiers of Research & Technology (QFort), National Cheng Kung University, Tainan 70101, Taiwan*

[7] *Meta-nanoPhotonics Center, National Cheng Kung University, Tainan 70101, Taiwan*

[*]Corresponding Authors**,** E-mails: mkchen@cityu.edu.hk; pcwu@gs.ncku.edu.tw; shumin.xiao@hit.edu.cn; dptsai@cityu.edu.hk

[†]These authors contributed equally to this work.

Supplementary Note 1: Refractive index of silicon

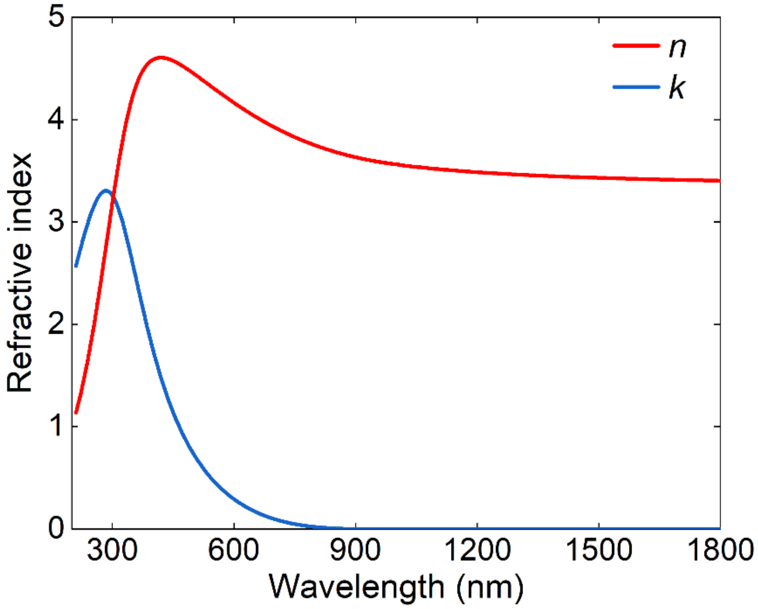

Supplementary Fig. 1: The refractive index of silicon film obtained through the optical measurement using an ellipsometer.

Supplementary Note 2: Fabrication process

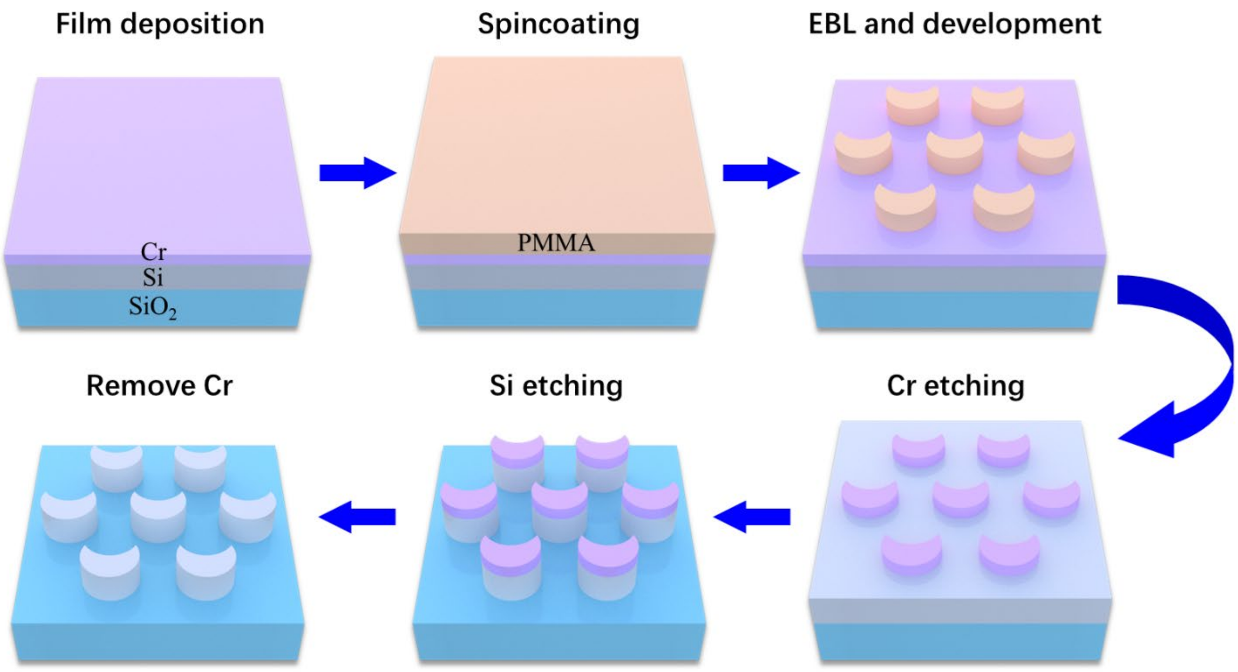

Supplementary Fig. 2: The fabrication process of the silicon meta-lens.

Supplementary Note 3: Measurement setups

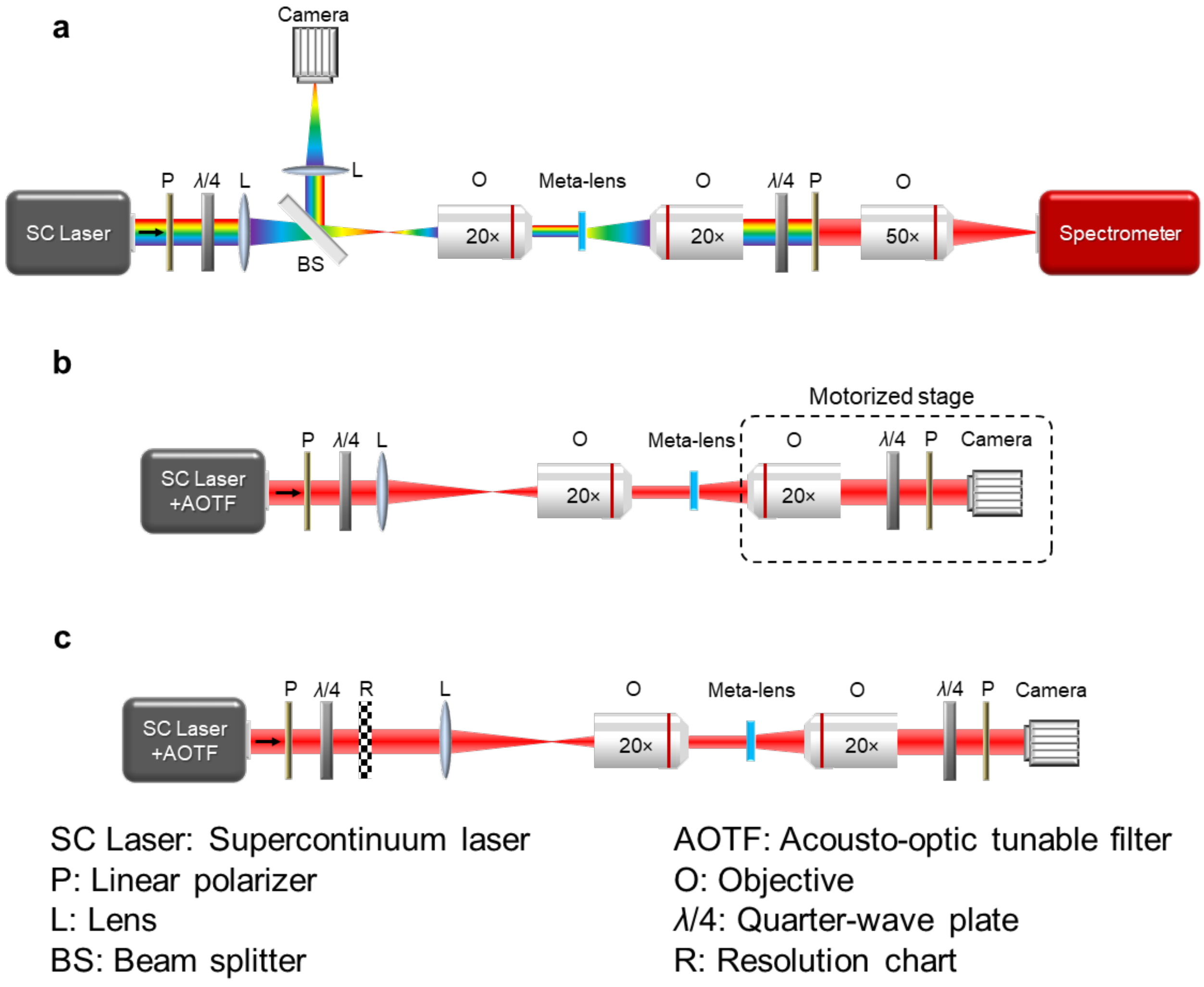

Supplementary Fig. 3: **a**,**b**,**c**, Optical measurement setups for transmission spectrum (**a**), light intensity distribution (**b**), and imaging (**c**). Supercontinuum laser is used to provide wideband coherent light. The circular polarization is generated by using a linear polarizer and a quarter-wave plate. A lens and an object (20× magnification, NA = 0.4) are employed to collimate the incident light. An object (20× magnification, NA = 0.4) is used to collect the transmission light. After passing through the quarter-wave plate and the linear polarizer, light intensity corresponding to LCP and RCP components can be analyzed by spectrometer or camera.

Supplementary Note 4: Simulated spectra of three integrated-resonant units

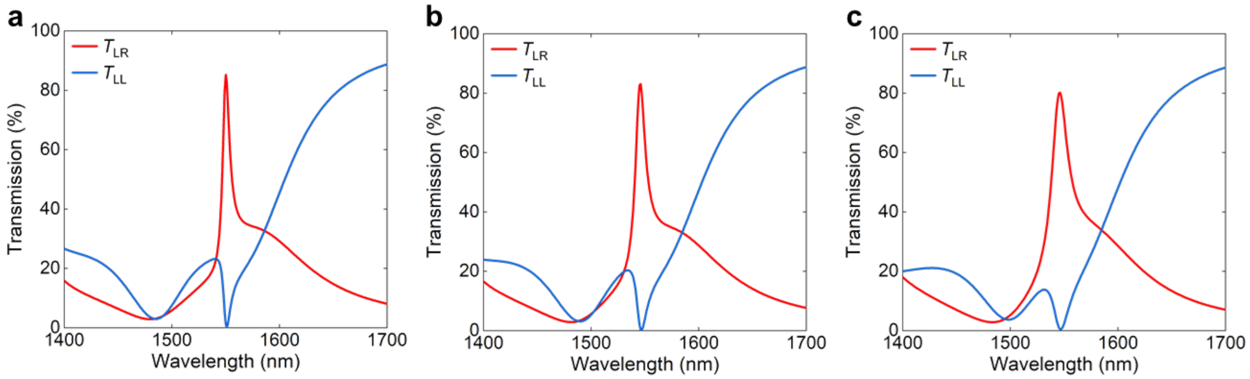

Supplementary Fig. 4: a-c, Simulated transmission spectra of three periodic integrated-resonant units (IRUs), corresponding to three samples shown in Fig. 3 in the main text.

Considering the arrangement of meta-atoms with different orientations in corresponding positions within the meta-lens, it is crucial to evaluate the rotation robustness of these meta-atoms, as demonstrated in Fig. 2g. This property indicates that the resonance attributes of meta-atoms, including resonant wavelength, efficiency, and phase shift, remain constant across varying rotation angles. Consequently, the nonlocal effect of interactions between meta-atoms is minimally affected, leading to a meta-lens akin to the designed periodic array. Nevertheless, despite the careful design of units, the meta-lens still experiences performance reduction compared to the inherent nature of the nonlocal effect. This difference is evident in the comparison between Fig. 3d-f and Supplementary Fig. 4. The simulated Q-factors and efficiencies of three meta-lens (periodic array of units) are 120 (205), 110 (144), and 71 (89), with corresponding efficiencies of 70% (85%), 72% (83%), 69% (80%), respectively. Further optimization of performance may be achieved by leveraging artificial intelligence technology to refine the interactions between meta-atoms with different orientations.

Supplementary Note 5: Transmission spectra with different offsets

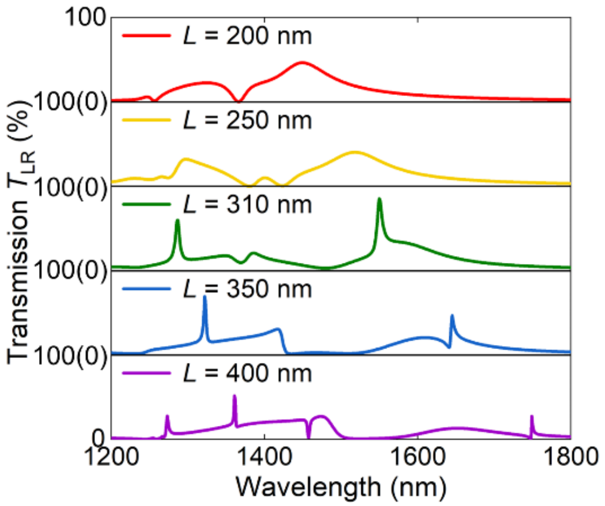

Supplementary Fig. 5: Transmission spectra with offset L = 200, 250, 310, 350, and 400 nm.

Supplementary Note 6: Demonstration of the nonlocal effect

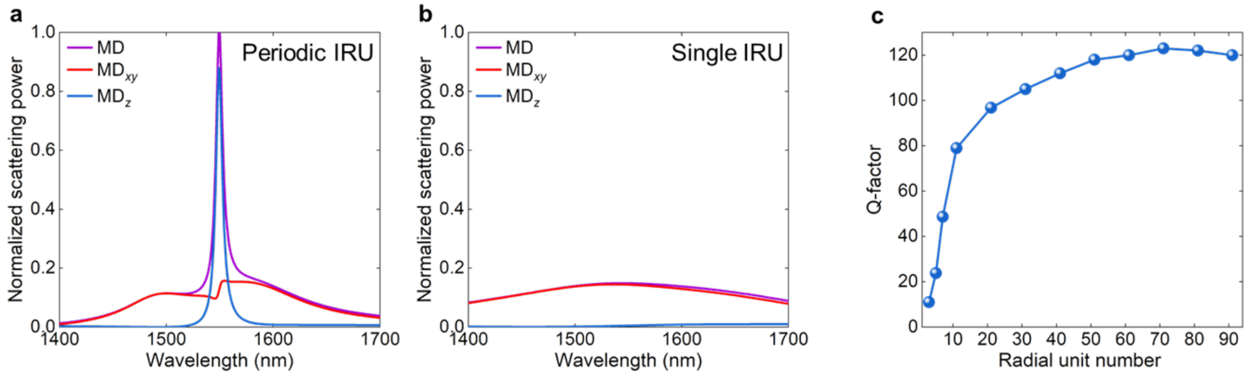

Supplementary Fig. 6: Demonstration of the nonlocal effect. a,**b**, Multipole decompositions of the periodic IRU (**a**) and the single IRU (**b**). **c**, Dependence of the Q factor on the number of meta-units along the radial axis.

To demonstrate the nonlocal effect in the proposed nonlocal Huygens' meta-lens, Supplementary Fig. 6a and b show the multipole decompositions of periodic IRU and single IRU, respectively. Low-Q Mie resonance (MD_{xy}) and high-Q q-BIC mode (MD_z) can both be excited in the period IRU. For a single IRU, without the nonlocal effect provided by the coupling with adjacent units, only the local Mie resonance can be excited. The relationship between the Q-factor and the number of meta-units along the radial axis in the meta-lens is provided in Supplementary Fig. 6c. It is evident that the Q-factor rises with the increase in the number of meta-units along the radial direction, highlighting the presence of a nonlocal effect within the meta-lens, even when the building blocks possess different structural orientations. The Q-factor increases as the number of meta-units grows, and it stabilizes at ~120 when the unit count exceeds 50. This further confirms the presence of the nonlocal effect and provides guidance for determining the meta-lens size.

Supplementary Note 7: Discussion on the theoretical limit in nonlocal meta-lens

As discussed in the main context, the metasurface can be conceptualized as a four-port system. In such cases, the transmission coefficient of cross-polarization conversion t_{LR} can be derived as[1,2]

$$\left|t_{\mathrm{LR}}\right|^2 = \mathrm{Re}\left[t_{\mathrm{LL}}\right] - \left|t_{\mathrm{LL}}\right|^2 \tag{1}$$

Here, t_{ij} represents the transmission coefficient for i-polarized light when the metasurface is illuminated with j-polarized light. The subscripts *L* and *R* signify left-handed polarization and right-handed polarization, respectively. Referring to Supplementary Equation (1), the maximum cross-polarization conversion efficiency $T_{LR} = |t_{LR}|^2$ reaches 25% when $t_{LL} = 50\%$.

Supplementary Note 8: Demonstration of contribution from Kerker effect

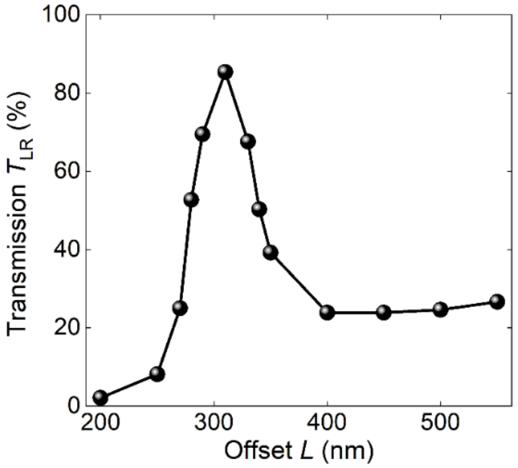

Supplementary Fig. 7: Transmission T_{LR} of q-BICs at resonant wavelengths as a function of offset L.

In the realm of dielectric metasurfaces, disrupting out-of-plane symmetry has been identified as a means to surpass the 25% efficiency threshold. In fact, two scenarios meeting this criterion have been explored. The first scenario involves breaking out-of-plane symmetry, which arises from the difference in refractive index between the meta-atoms and the substrate. To exceed the 25% conversion efficiency threshold using this approach, activation of resonances in the substrate like guide-mode resonance is necessary[3]. However, achieving precise control over the phase of each meta-atom poses a significant challenge, as resonance occurs both at the individual meta-atom level and within guided waves, with the latter restricting individual phase modulation. Alternatively, in the second scenario, the height of meta-atoms in local dielectric meta-lenses typically needs to match the wavelength level[4]. This configuration facilitates the generation of waveguide-like modes inside the high-index meta-atoms, enabling precise phase control using individual units and overcoming limitations on polarization conversion efficiency (see the left panel in Fig. 1).

In this work, we abstain from introducing guide-mode resonance within the substrate or utilizing meta-atoms with wavelength-level height. Consequently, our proposed approach does not hinge on breaking out-of-plane symmetry to surpass the 25% polarization conversion efficiency threshold. Furthermore, the predominance of vertical magnetic dipole (MD) moments in the quasi-bound state in the continuum (q-BIC) mode, primarily contributed by the in-plane current, lends support to our claim: the disruption of out-of-plane symmetry in our design is minimal. To further illustrate that our proposed approach for achieving >25% polarization conversion efficiency in an optically thin metasurface is attributable to the involvement of both the MD and q-BIC, we present the polarization conversion efficiencies T_{LR} for various offsets L that control the spectral separation between MD and q-BIC. As can be seen in Fig. 2b in the main article and Supplementary Fig. 7, when the offset L ranges between 270 nm and 400 nm, the Mie-type MD resonance couples with the q-BIC mode, resulting in the generalized Kerker effect. Consequently, the transmission polarization conversion efficiency exceeds 25%, reaching a maximum of 85.4% with L = 310 nm. However, when L exceeds 400 nm or falls below 270 nm, the two resonant wavelengths are significantly distant, and the single mode of q-BIC can only achieve an efficiency limited to approximately 25%. If the designed unit possesses an intrinsically large out-of-plane asymmetry, the efficiencies for all offsets should not be limited to 25%, and the rapid increase around L = 310 nm would not occur. These observations confirm the intrinsic weak out-of-plane asymmetry of the designed unit and underscore the importance of the Kerker effect.

Supplementary Note 9: Design process of IRU

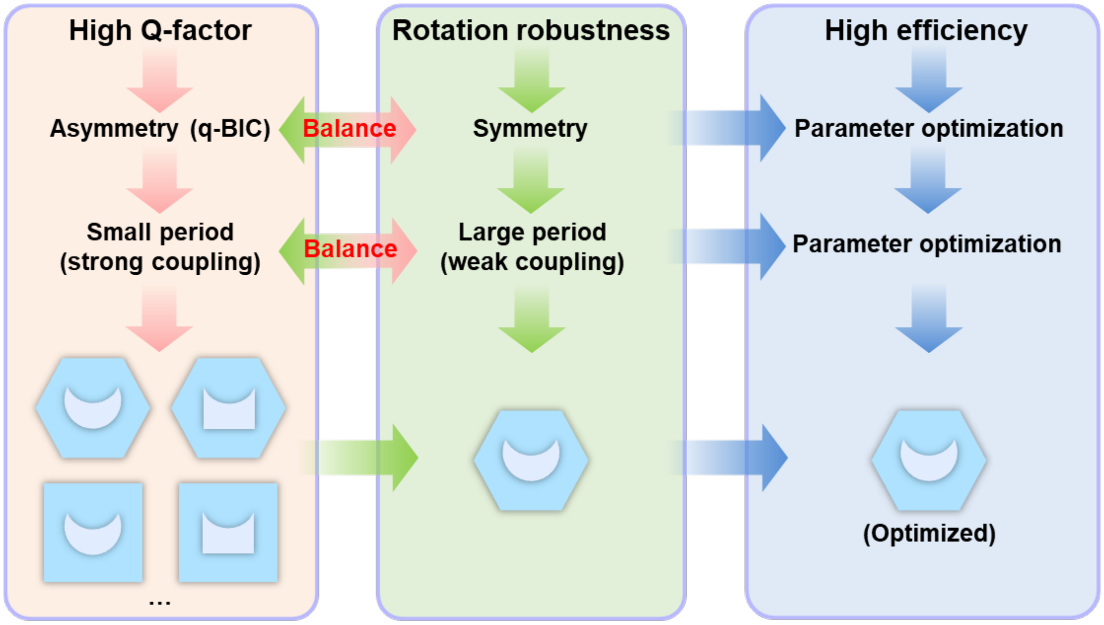

Supplementary Fig. 8: Design flow for an IRU with high Q-factor, rotation robustness, and high efficiency.

In the main article, we delve into the realm of meta-lenses, particularly focusing on the widespread exploration of broadband varieties with local responses. These lenses, prized for their versatility, often boast straightforward designs and polarization control, thanks to the independent manipulation of individual meta-atoms. However, our attention shifts to narrowband meta-lenses with nonlocal responses, tailored for specific applications. Herein lies the challenge: striking a delicate balance between harnessing individual meta-atom responses for local phase control and navigating the complexities of nonlocal resonance excitation arising from interactions among neighboring meta-atoms. Presently, no established strategies exist to comprehensively address the myriad facets - Q-factor, efficiency, manipulation dimension, and footprint - simultaneously within the context of nonlocal wavefront shaping. Our work endeavors to tackle this conundrum with a novel approach, exemplified by a meticulously crafted metasurface. Let us delve deeper

into the pioneering aspects and advancements of our methodology on the pioneering design of IRU (also refer to Supplementary Fig. 8).

(a) Amplified Q-factor: By disrupting the structure's in-plane symmetry, we usher in symmetry-protected q-BICs, lending a significant boost to the Q-factor. This augmentation is achieved by fine-tuning the asymmetric parameter, affording flexibility in manipulation.

(b) Stalwart rotation angle robustness: The rotation angle assumes paramount importance in generating the geometric phase essential for meta-lens functionality. Ensuring the steadfastness of resonance characteristics - Q-factor, efficiency, phase, and resonant wavelength - across varying rotation angles is imperative for seamless phase modulation and resonance excitation.

(c) Enhanced transmission efficiency: Beyond the q-BIC mode, our innovation encompasses the incorporation of a Mie-type MD resonance. This synergistic coupling with q-BIC engenders the generalized Kerker effect, fostering unidirectional transmission and surpassing the theoretical limit of 25% for transmissive circular polarization conversion.

Concurrently attaining these three pivotal points presents a formidable challenge within the realm of conventional meta-units characterized by a single resonant mode and lackluster structure. Moreover, the optimization of IRUs warrants a delicate equilibrium, wherein geometric shape

optimization necessitates a harmonious interplay between symmetry and asymmetry to cater to rotation robustness and the excitation of leaky q-BIC excitation. Selecting a lattice and unit with improved symmetry while maintaining asymmetry, such as a hexagonal lattice, optimizes this balance. The crescent shape, derived from a circle, is chosen for its superior symmetry, and the optimization process is detailed in Supplementary Fig. 9. Similarly, the optimization of array periods demands a judicious equilibrium, balancing weak coupling for rotation robustness with strong coupling between IRUs to realize a high Q-factor (refer to Supplementary Fig. 10).

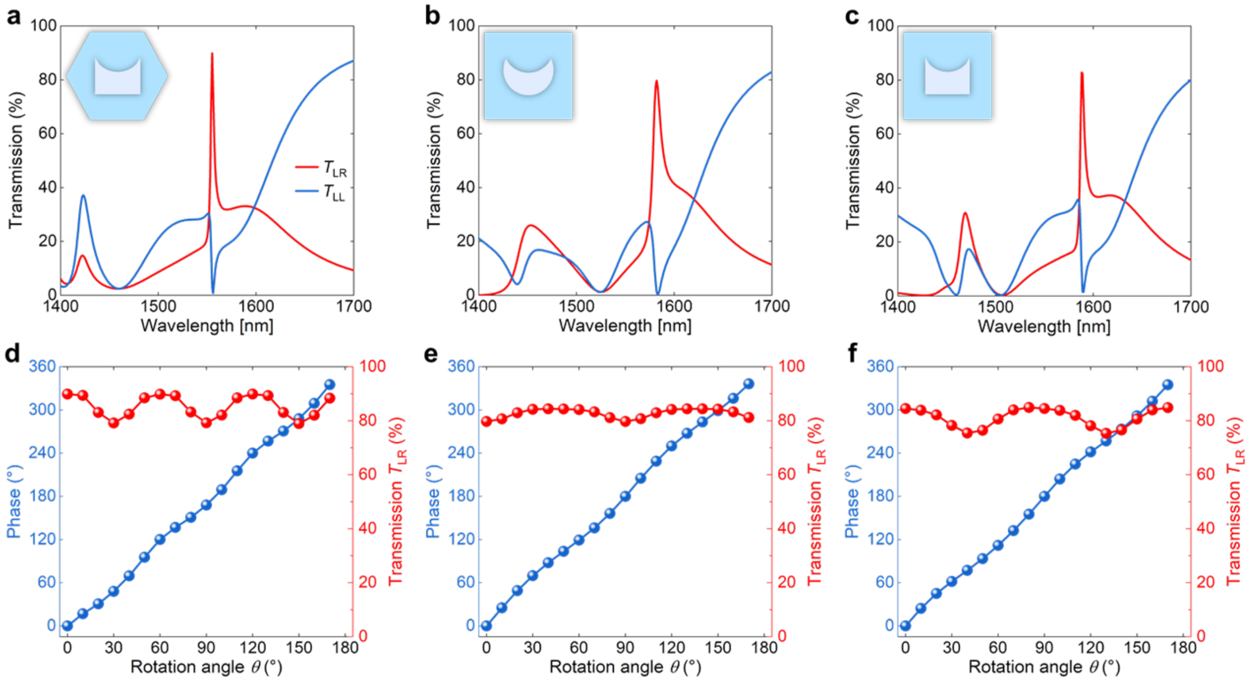

Supplementary Fig. 9: (**a-c**) Transmission spectra and (**d-f**) dependences of efficiency and phase on the rotation angle for three IRUs with different geometries. Insets are their schematic diagrams. Array periods are all 1000 nm. The length of squares and diameter of circles are both 620 nm.

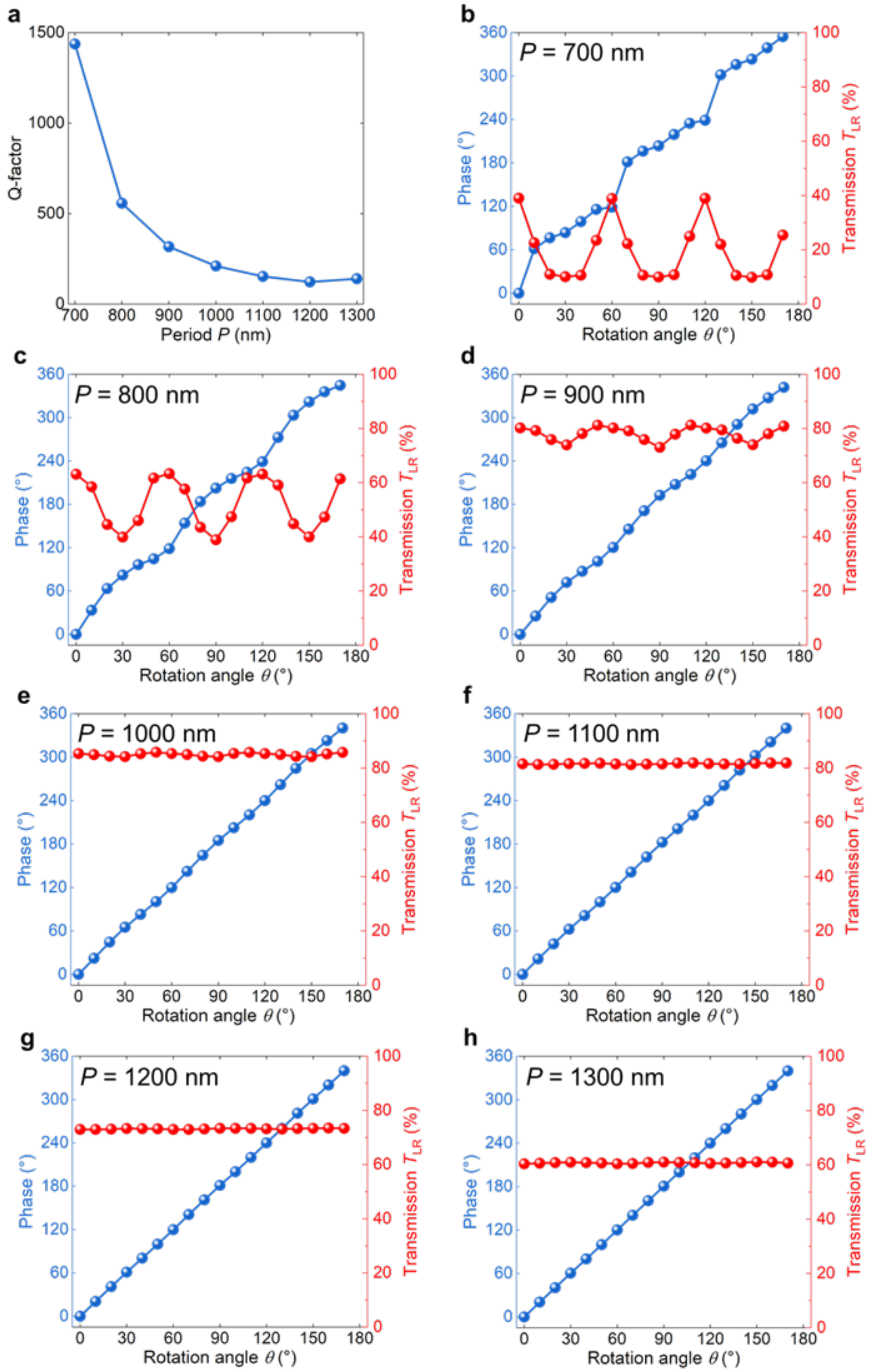

Supplementary Fig. 10: a, Q-factor as a function of period *P*. **b-h**, Dependences of phase and transmission T_{LR} on the rotation angle of IRU for periods *P* varying from 700 nm to 1300 nm.

Supplementary Note 10: Scattering spectra of horizontal multipoles

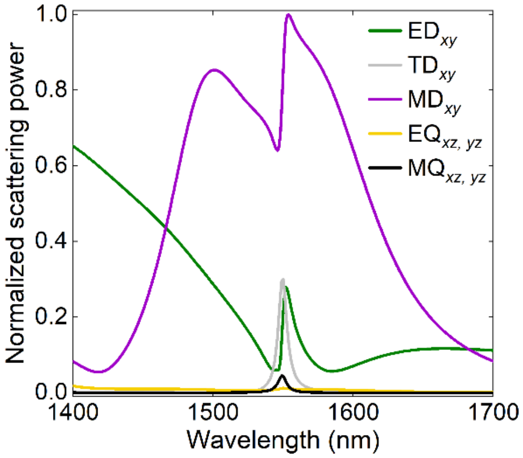

Supplementary Fig. 11: Normalized scattering power of horizontal multipoles of an IRU with L = 310 nm.

The vertical MD (MD_z) resonance (q-BIC) is originally a dark mode for normal incidence. By breaking the symmetry in the parameter space, the q-BIC mode is effectively induced by asymmetric horizontal dipole moments. The vertical MD cannot generate vertical radiation, but other horizontal multipole moments induced by the q-BIC mode can influence the normal transmission, such as electric dipole, toroidal dipole, etc., which can be found by the peak in the scattering spectra of horizontal multipoles in Supplementary Fig. 11. These multipoles interfere with the horizontal MD induced by the Mie-type MD resonance, generating the generalized Kerker effect to break the radiation symmetry and improve the transmission efficiency.

Supplementary Note 11: Influence of round corners

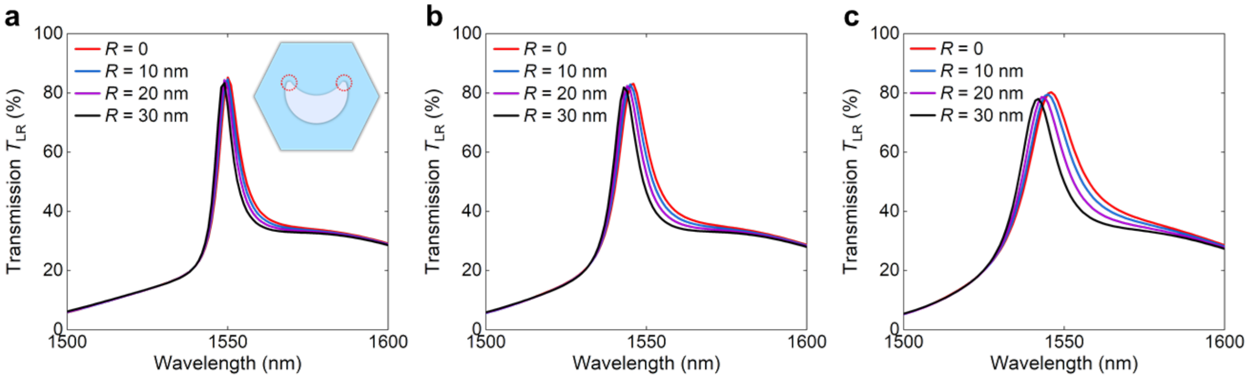

Supplementary Fig. 12: a-c, Simulated transmission spectra T_{LR} of three IRUs with the radius of round corners R = 0, 10, 20, 30 nm (red circles in the inset).

Supplementary Note 12: Measured spectra by uncollimated incoherent light

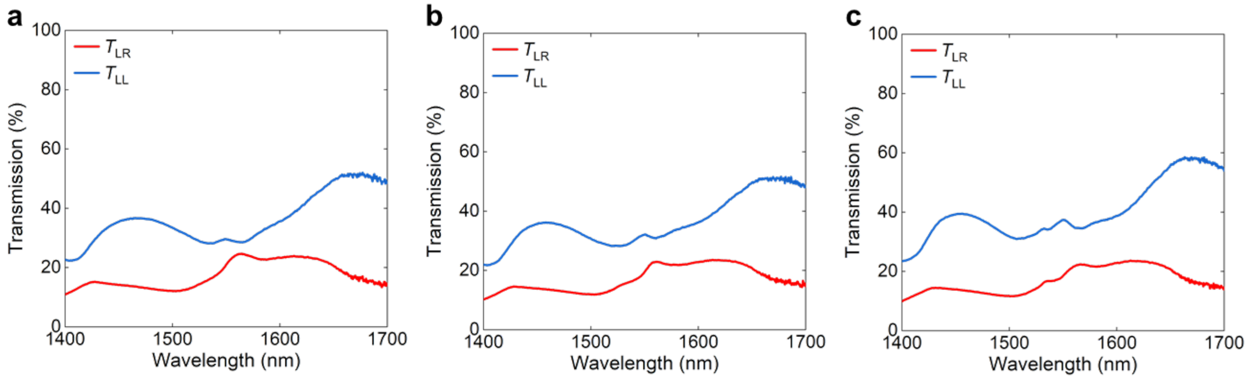

Supplementary Fig. 13: a-c, Measured spectra of three nonlocal Huygens' meta-lenses illuminated by uncollimated incoherent light.

Supplementary Fig. 13 represents the measured spectra of three nonlocal Huygens' meta-lenses illuminated under ambient conditions of uncollimated incoherent light (OCTW-UV-VIS-NIR from Oceanhood). Despite the presence of two resonances in the spectra, the Q-factor and efficiency performances of the nonlocal meta-lens are diminished. Addressing the dependence on collimation and coherence phenomena requires additional design and optimization of both local and nonlocal responses. This optimization is necessary to enhance the effectiveness of the meta-lens in operating within natural environments.

Supplementary Note 13: Simulated focusing profiles

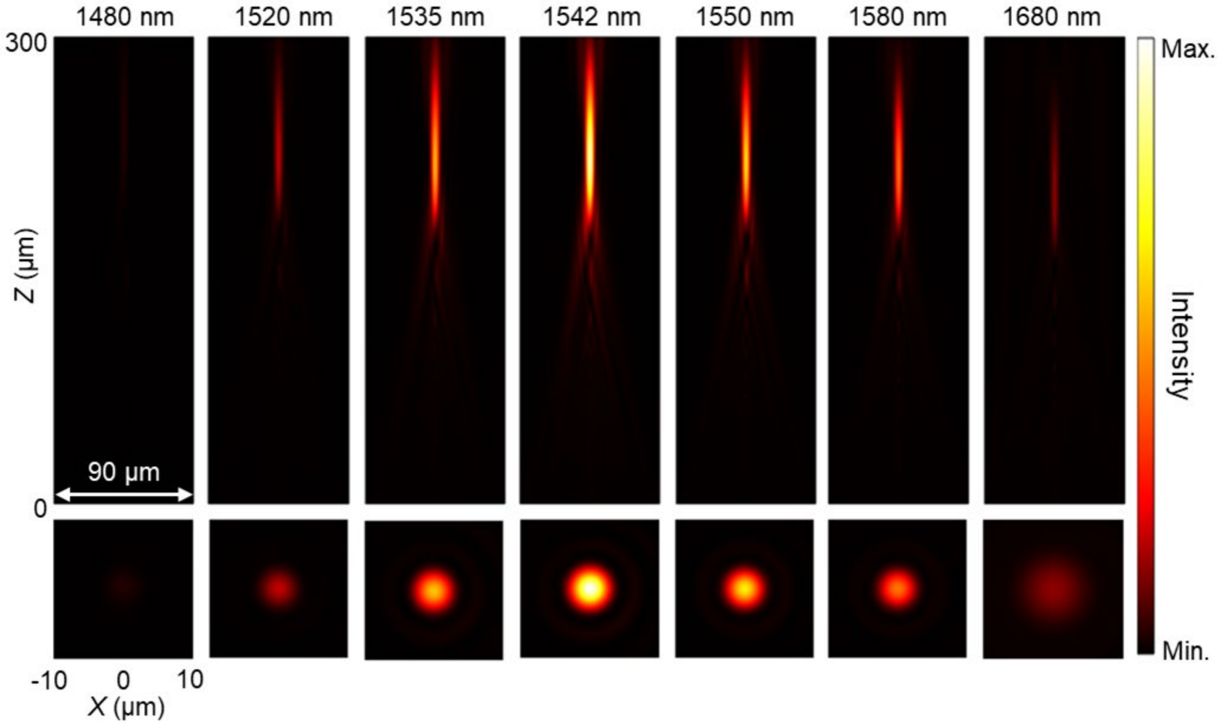

Supplementary Fig. 14: Simulated *xz*- and *xy*-plane intensity distributions of the nonlocal Huygens' meta-lens at different wavelengths.

The experimental resonant wavelength undergoes a red shift of ~20 nm compared with the simulated wavelength, which is primarily due to the fabrication imperfection. Figure 2b demonstrates that the excitation of q-BIC mode remains relatively stable, even with slight deviations in feature size, resulting in only a slight shift of resonant wavelength and a minor change of Q-factor. Another crucial observation is that the Kerker effect appears more resilient to fabrication imperfections as the Mie-type MD resonance exhibits a low Q-factor, leasing only to a wavelength shift and a slight reduction in efficiency. Consequently, only a wavelength deviation of about 20 nm occurs, while the overall phenomenon of wavelength-selective property remains minimally affected, as evident in Fig. 4e and Supplementary Fig. 14.

Supplementary Note 14: Performance comparison

Supplementary Table 1. Performance comparison between meta-devices for narrowband wavefront shaping.

References	Quality-factor	Efficiency	Wavefront shaping dimension	Number of layer
This work (Exp.)	104	55%	2D	1
This work (Sim.)	120	70%	2D	1
Ref. 5 (Exp.)	~86	~4%	2D	1
Ref. 6 (Exp.)	>2500	>20%	1D	1
Ref. 7 (Exp.)	~380	59%	1D	1
Ref. 8 (Sim.)	~120	~25%	2D	1
Ref. 9 (Sim.)	~500	>90%	2D	2
Ref. 10 (Sim.)	~500	>90%	2D	2
Ref. 11 (Sim.)	~3600	~60%	1D	1
Ref. 12 (Sim.)	~30000	>80%	1D	1

We have compared our work with Ref. [5] in the main text because both two works are single-layer nonlocal meta-lenses for two-dimensional wavefront shaping. To provide a more comprehensive comparison, a comparison with another similar work of Ref. [7] is also given as follows. It proposed a novel phase gradient metasurface made entirely from individually addressable high-Q-factor meta-atoms. Although the Q-factor of ~380 reported in Ref. [7] is higher than our work and the efficiency of 59% is comparable, it is important to highlight the advantages and distinctions of our work as follows:

(a) Advantages

i. Our work achieves two-dimensional wavefront shaping with high Q-factor and efficiency, surpassing the previous work's demonstration of only one-dimensional control[7]. The

addition of wavefront shaping dimension significantly impacts optical performance, while the limited manipulation dimension constrains versatile applications.

ii. Unlike previous work, our fabrication process doesn't demand extreme precision. In Fig. 2b in the main article, the q-BIC mode and Kerker effect exhibit stability even with slight wavelength deviations in changing geometric parameters. In contrast, previous work requires precise control over geometric parameters for wavefront engineering[5], posing challenges for practical implementation due to the need for a fabrication process with precision up to a few nanometers.

iii. Transmissive optical components are more commonly used in practical applications. The involvement of a metallic back reflector in Ref. [7] confines the metasurface to operate only in a reflection scheme, limiting its range of applications. In contrast, our metasurface operates in a transmission scheme with 2D wavefront control capability, making it more promising for real-world applications.

(b) Differences

The physical mechanisms between our work and previous work are inherently distinct. In our approach, we leverage q-BIC and the Kerker effect to achieve both high Q-factor and efficiency, along with optimized rotation robustness for the geometric phase. In contrast, the reported work manipulates the geometric parameters of meta-atoms to control the resonance phase of guide-mode resonances. Additionally, the incident polarization states differ, with our work utilizing circular polarization and the reported work employing linear polarization. Despite these differences, both approaches offer a platform for efficient high-Q wavefront shaping.

Supplementary Note 15: Multispectral nonlocal Huygens' meta-lens in visible

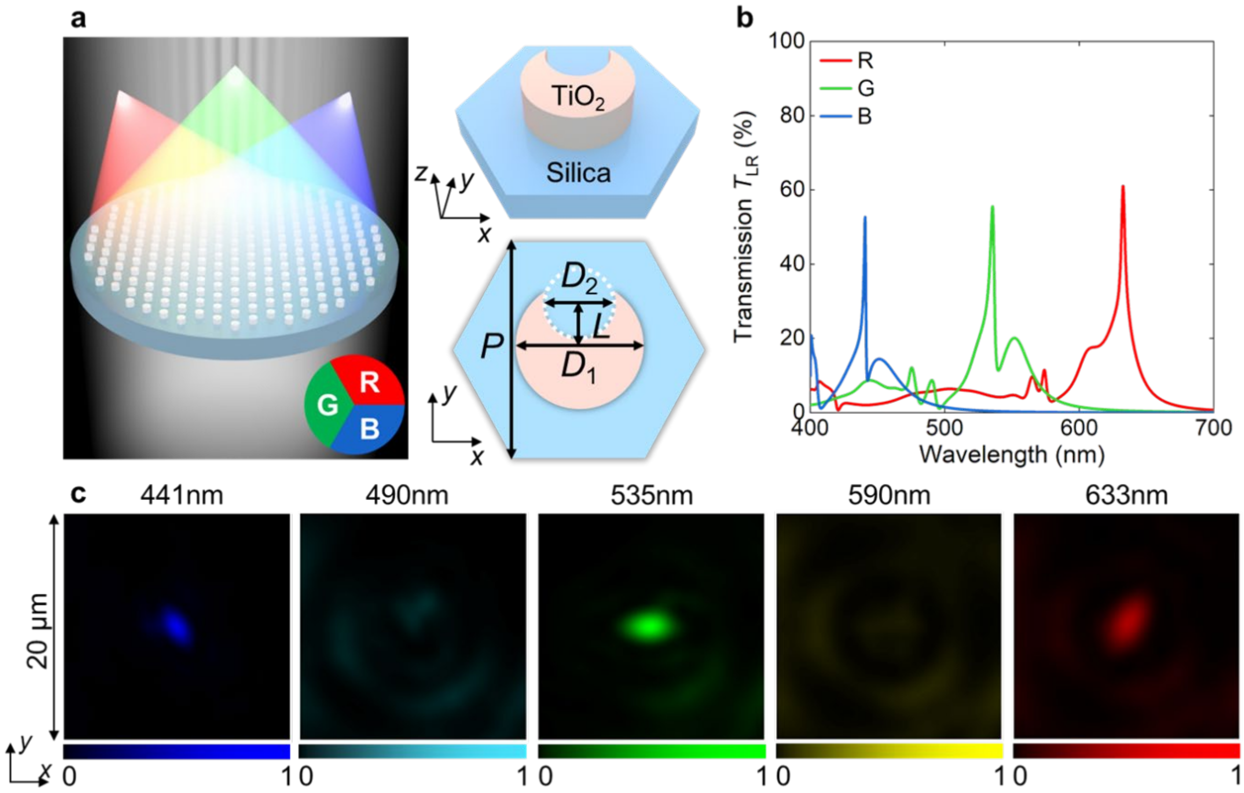

Supplementary Fig. 15: Multispectral nonlocal Huygens' meta-lens in the visible. a, Schematic diagram. The meta-lens is divided into three areas for three colors. **b**, Transmission spectra T_{LR} of three IRU operating at wavelengths 441, 535, and 633 nm. **c**, Intensity distributions in the *xy* plane at different wavelengths.

The proposed nonlocal Huygens' meta-lens can be extended to the visible band, providing potential applications in multispectral color routers and augmented reality/virtual reality displays, as shown in Supplementary Fig. 15. Furthermore, the nonlocal Huygens' meta-lens holds promise in nonlinear generation and biomedical imaging, surpassing the capability of conventional or broadband meta-lenses. For example, nonlinear meta-lenses need both high-Q-factor resonance and phase modulation capability, while biomedical imaging demands specific wavelengths to match the spectral responses of biomolecules.

Supplementary References

1. Ding X., Monticone F., Zhang K., Zhang L., Gao D., Burokur S. N., De Lustrac A., Wu Q., Qiu C. W., Alù A. Ultrathin Pancharatnam–Berry metasurface with maximal cross-polarization efficiency. *Adv. Mater.* **27**, 1195-1200 (2015).

2. Monticone F., Estakhri N. M., Alu A. Full control of nanoscale optical transmission with a composite metascreen. *Phys. Rev. Lett.* **110**, 203903 (2013).

3. Semnani B., Flannery J., Al Maruf R., Bajcsy M. Spin-preserving chiral photonic crystal mirror. *Light: Sci. Appl.* **9**, 23 (2020).

4. Wang S., Wu P. C., Su V.-C., Lai Y.-C., Chen M.-K., Kuo H. Y., Chen B. H., Chen Y. H., Huang T.-T., Wang J.-H., Lin R.-M., Kuan C.-H., Li T., Wang Z., Zhu S., Tsai D. P. A broadband achromatic metalens in the visible. *Nat. Nanotechnol.* **13**, 227-232 (2018).

5. Malek S. C., Overvig A. C., Alu A., Yu N. Multifunctional resonant wavefront-shaping meta-optics based on multilayer and multi-perturbation nonlocal metasurfaces. *Light: Sci. Appl.* **11**, 246 (2022).

6. Lawrence M., Barton III D., Dixon J., Song J. H., van de Groep J., Brongersma M. L., Dionne J. A. High quality factor phase gradient metasurfaces. *Nat. Nanotechnol.* **15**, 956-961 (2020).

7. Lin L., Hu J., Dagli S., Dionne J. A., Lawrence M. Universal narrowband wavefront shaping with high quality factor meta-reflect-arrays. *Nano Lett.* **23**, 1355-1362 (2023).

8. Overvig A. C., Malek S. C., Yu N. Multifunctional Nonlocal Metasurfaces. *Phys. Rev. Lett.* **125**, 017402 (2020).

9. Overvig A., Yu N., Alù A. Chiral Quasi-Bound States in the Continuum. *Phys. Rev. Lett.* **126**, 073001 (2021).

10. Overvig A., Alù A. Wavefront-selective Fano resonant metasurfaces. *Adv. Photonics* **3**, 026002 (2021).

11. Klopfer E., Lawrence M., Barton III D., Dixon J., Dionne J. A. Dynamic Focusing with High-Quality-Factor Metalenses. *Nano Lett.* **20**, 5127-5132 (2020).

12. Klopfer E., Dagli S., Barton III D., Lawrence M., Dionne J. A. High-quality-factor silicon-on-lithium niobate metasurfaces for electro-optically reconfigurable wavefront shaping. *Nano Lett.* **22**, 1703-1709 (2022).
